# The Role of AGE/RAGE Signaling in Diabetes-Mediated Vascular Calcification

**DOI:** 10.1155/2016/6809703

**Published:** 2016-07-28

**Authors:** Amber M. Kay, C. LaShan Simpson, James A. Stewart

**Affiliations:** ^1^Department of Biological Sciences, Mississippi State University, Mississippi State, MS 39762, USA; ^2^Department of Agricultural and Biological Engineering, Mississippi State University, Mississippi State, MS 39762, USA

## Abstract

AGE/RAGE signaling has been a well-studied cascade in many different disease states, particularly diabetes. Due to the complex nature of the receptor and multiple intersecting pathways, the AGE/RAGE signaling mechanism is still not well understood. The purpose of this review is to highlight key areas of AGE/RAGE mediated vascular calcification as a complication of diabetes. AGE/RAGE signaling heavily influences both cellular and systemic responses to increase bone matrix proteins through PKC, p38 MAPK, fetuin-A, TGF-*β*, NF*κ*B, and ERK1/2 signaling pathways in both hyperglycemic and calcification conditions. AGE/RAGE signaling has been shown to increase oxidative stress to promote diabetes-mediated vascular calcification through activation of Nox-1 and decreased expression of SOD-1. AGE/RAGE signaling in diabetes-mediated vascular calcification was also attributed to increased oxidative stress resulting in the phenotypic switch of VSMCs to osteoblast-like cells in AGEs-induced calcification. Researchers found that pharmacological agents and certain antioxidants decreased the level of calcium deposition in AGEs-induced diabetes-mediated vascular calcification. By understanding the role the AGE/RAGE signaling cascade plays diabetes-mediated vascular calcification will allow for pharmacological intervention to decrease the severity of this diabetic complication.

## 1. Introduction

Diabetes mellitus is a family of diseases characterized by elevated blood glucose levels or hyperglycemia resulting from the body's inability to produce and/or use the insulin hormone. Type I diabetes mellitus is associated with pancreatic *β* cell dysfunction resulting in the loss of insulin production, whereas type II diabetes mellitus is caused by insulin receptor dysfunction in which insulin receptor signaling is uncoupled from glucose uptake. Diabetes mellitus is highly prevalent in the United States with approximately 29 million people living with diabetes or 9.3% of the population [1]. It is reported that the death rate from cardiovascular disease for an individual, 18 years and older, with diabetes was about 1.7 times higher than the normal population [1]. Increased death rates from diabetic cardiovascular disease demonstrate the severity of the complications that can arise from this pathology. Therefore, the link between cardiovascular disease and diabetes is essential to understand [2].

## 2. Type II Diabetes and Vascular Calcification

Type II diabetes has been heavily linked to vascular calcification through several different mechanisms, some of which include oxidative stress, hyperglycemia, hyperkalemia, and hypercalcemia with oxidative stress being the main focus of this review [3–5]. Vascular calcification is described as the hardening of the medial layer of the artery through deposition of hydroxyapatite minerals into the extracellular matrix [6–8]. This process, once thought to be passive and associated with aging, has now been demonstrated to be a tightly regulated cell-mediated process [3]. During vascular calcification, bone morphogenetic protein-2 (BMP-2) activates core binding factor alpha-1 (CBFA-1, also known as RunX2), which acts as the primary transcriptional regulator for the maturation of osteoblasts in the bone [9–11]. CBFA-1 also upregulates the production of osteoblast proteins within vascular smooth muscle cells (VSMCs), which is thought to cause a phenotypic switch of VSMCs to an osteoblast-like phenotype [12]. Alkaline phosphatase (ALP) and bone sialoprotein (BSP) have been demonstrated to be early markers of osteoblast activity, while markers, such as osteopontin (OPN) and osteocalcin, are upregulated late in the calcification process [13–15]. Their primary function is to enhance the formation and deposition of hydroxyapatite, which is composed of type I collagen and other noncollagenous proteins [15]. Primarily indicated in bone formation, ALP is responsible for cleaving pyrophosphate to phosphate to promote hydroxyapatite deposition and mineralization within the bone [16]. BSP is responsible for the nucleation of hydroxyapatite mineral [15, 17, 18]. Similar to ALP, OPN is also linked to hydroxyapatite deposition and can serve as a mediator of cell attachment and signaling [19]. Hydroxyapatite size and shape are mediated by osteocalcin through a vitamin K dependent mechanism [20]. Taken together, these data demonstrate the potential to promote bone formation within a living system, and researchers have utilized this knowledge of bone matrix proteins to understand the underlying mechanisms of vascular calcification and type II diabetes.

In a series of studies performed by Chen et al., arteries harvested from diabetic and nondiabetic patients were analyzed to determine the amount of calcium, OPN, ALP, type I collagen, and BSP. With the exception of BSP, all investigated bone matrix proteins were significantly increased as a result of diabetes [21].* In vitro *experiments, using bovine vascular smooth muscle cells (BVSMCs) grown in euglycemic (normal glucose) and hyperglycemic conditions, revealed that CBFA1, ALP, and osteocalcin levels were significantly higher in cells grown in a high glucose media. In addition, calcium deposition was also significantly higher in high glucose than in normal glucose media, and this trend was also observed when both types of growth media conditions were supplemented with calcification media. Calcification media contain elevated levels of inorganic phosphate to promote calcification through utilization of the cells that need to maintain homeostasis. To determine the signaling mechanisms responsible for the increased bone matrix protein expression, BVSMCs were exposed to high glucose levels and protein kinase C (PKC) activity was pharmacologically inhibited in both normal and high glucose treated cells. PKC was selected as the signaling pathway focus due to its predetermined role in cellular responses to diabetes and hyperglycemia [22, 23]. As a result, the expression of bone matrix proteins was significantly decreased, whereas, in normal glucose treated cells, there was no notable change in protein expression. This study also demonstrated enhanced BMP-2 secretion from BVSMCs cultured in high glucose media. Overall, Chen et al. concluded that hyperglycemic conditions, as observed in diabetes, promoted the upregulation of bone matrix proteins and vascular calcification [21, 24]. Supporting studies by Mori et al. demonstrated OPN was upregulated and activated by a similar PKC-mediated pathway in diabetic rat VSMCs. Western blotting confirmed that PKC inhibition resulted in a notable decrease in OPN protein expression [25–27]. Taken together, these studies have shown not only the prevalence of bone matrix protein expression in vascular smooth muscle cells but also the role of PKC in diabetes-mediated vascular calcification.

## 3. Vascular Calcification and AGE-RAGE Signaling

In addition to increased bone matrix protein expression in VSMCs during diabetic and calcification treatments, studies have also shown that advanced glycation end products (AGEs) and their receptors (RAGEs) play a role in vascular calcification [28]. Type II diabetes patients have been shown to have a significantly higher concentration of AGEs than the nondiabetic population [29–31]. AGEs form over a lifetime as a result of increased circulating glucose as well as other reducing sugars, such as galactose and fructose, reacting with amino groups of proteins to form Schiff bases to either follow the polyol pathway to yield AGEs or be degraded [32]. These glycated end products interact with RAGEs, which are transmembrane proteins that are a part of the immunoglobulin superfamily. RAGEs are upregulated in response to increased circulating AGE levels [33]. Upon AGE-RAGE binding, RAGE works through PKC-*ζ* to trigger the downstream activation of a signaling cascade that works through p38 mitogen activated protein kinase (MAPK), transforming growth factor-*β* (TGF-*β*), and nuclear factor *κ*B (NF*κ*B) [34, 35]. Suga et al. demonstrated that activation of the AGE-RAGE signaling in rat VSMCs reduced the expression of VSMC gene markers such as smooth muscle-myosin heavy chain (SM-MHC) and smooth muscle 22*α* (SM22*α*) [36]. This downregulation of VSMCs markers suggests the possible phenotypic switch of VSMCs to an osteoblast-like phenotype [12]. This is supported by findings from human VSMCs (HVSMCs) where activation of RAGE increased mRNA expression and activity of ALP, a bone matrix protein, suggesting a role for RAGE signaling in vascular calcification [36]. These studies demonstrated some basic roles for RAGE in VSMC calcification through PKC-*ζ* signaling, increased expression of ALP, and decreased expression of VSMC gene markers.

In studies performed by Tanikawa et al., using an HVSMC* in vitro *calcification model increasing the levels of AGEs significantly increased the amount of calcium deposition after 7 and 14 days when compared to BSA treated and control samples [37]. Additionally, mRNA expression of CBFA-1 (RunX2), ALP activity, and osteocalcin protein levels were also significantly elevated. Together, these data indicated that AGE treatment promotes an osteoblast-like phenotype in HVSMCs. This phenotypic switching was not dependent on calcification media as similar results were found using HVSMCs grown with and without calcification media [21]. VSMC expression of osteoblast proteins may be linked to p38 MAPK activity as Tanikawa et al. found that, with increased AGE exposure, p38 MAPK activation was increased. Conversely, when RAGE signaling was dampened, p38 MAPK activation was decreased, and the changes in p38 MAPK correlated to decreased levels of ALP activity despite AGE-induced calcification [37]. In a similar study by Hu et al., p38 MAPK was shown to be essential for osteoblast differentiation in MC3T3-E1 cells. Pharmacological inhibition of p38 MAPK resulted in decreased in ALP activity, thus, demonstrating that p38 MAPK is required for ALP expression in osteoblast-like cells [38]. Therefore, ALP activity can be directly influenced by both increased AGE exposure and elevated RAGE cascade signaling through p38 MAPK. This relationship suggests that p38 MAPK plays a key role in the AGE-RAGE pathway in diabetes-mediated vascular calcification [37].

While these findings demonstrate the importance of the AGE-RAGE pathway in diabetes-mediated vascular calcification, Ren et al. demonstrated that AGEs also significantly increased intracellular calcium levels in rat VSMCs [37, 39, 40]. It was found that mRNA levels of ALP and OPN were significantly increased after a 24-hour exposure to glycated albumin (AGE-BSA). Due to the increase in ALP and OPN with AGE-BSA treatment, the group also demonstrated that RAGE was upregulated in the rat VSMCs. When incubated with a neutralizing antibody to RAGE, the amount of calcium and ALP expression was decreased. The observed changes confirmed that RAGE mediates AGE-induced VSMC calcification [39]. Wei et al. showed that diabetes accelerated aortic calcification in male Wistar rats [41]. The animals were treated with streptozotocin (STZ) to induce diabetes and then treated with Vitamin D3 and nicotine (VDN) to induce vascular calcification. von Kossa staining allowed for visualization of the calcium particles within the removed aortic tissue, and calcium particles were found within the selected tissue section. Western blot analysis showed a significant increase in ALP expression and the levels of AGEs were also increased in the diabetic and VDN treated animals [41]. It is important to point out that while AGE-RAGE signaling can directly mediate vascular calcification in diabetes, AGE-RAGE signaling can also indirectly impact this diabetic complication.

## 4. Roles for Fetuin-A in Vascular Calcification and RAGE Signaling

Serum protein *α*_2_-Heremans-Schmid glycoprotein (Ahsg or fetuin-A), a systemically circulating glycoprotein, has been implicated in insulin resistance in type II diabetic patients [42]. Patient data revealed that high levels of serum fetuin-A were an indicator for hyperglycemia in type II. Fetuin-A also hindered insulin reception through inhibition of the insulin receptor to autophosphorylate insulin receptor substrate-1 protein, which is crucial to the insulin receptor signaling pathway [43, 44]. Collectively, these studies revealed that fetuin-A plays a role in insulin resistance in type II diabetes which can lead to further exacerbation of hyperglycemia and other diabetic complications. Interestingly, increased levels of vascular calcification have been demonstrated to be associated with not only type II diabetes but also patients with chronic kidney disease (CKD) [45]. Vascular calcification, in this instance, has been shown to promote both inflammatory and oxidative stress response to compound it as a risk factor for cardiovascular disease. Fetuin-A is released by the liver to function as an acute phase protein in the innate immune system where it functions to promote anti-inflammatory and antioxidative stress responses to inhibit overexpressed inflammatory molecules.

Conversely, fetuin-A can also elicit an innate immune response elicited in part by toll-like receptors (TLRs). This mechanism can be activated by free fatty acids (FFAs) to induce a proinflammatory response [46]. Pal et al. showed that fetuin-A can act as a ligand to TLR-4 to stimulate FFA-induced insulin resistance in adipocytes [47]. In addition to promoting insulin resistance in type II diabetic patients, fetuin-A can also inhibit an alternate RAGE ligand, high mobility group box-1 (HMGB1), which is responsible for the release and recruitment of several cytokines, adhesion molecules, and chemokines. RAGE signal cascade activation has been demonstrated to be responsible for HMGB1 mediated expression of tumor necrosis factor (TNF) and interleukin-1 (IL-1) [48, 49]. Of concern, fetuin-A inhibition of HMGB1 could possibly create a setting for RAGEs to preferentially select and bind AGEs to activate the cascade. Using data collected from CKD patient samples, Janda et al. demonstrated that increased serum fetuin-A levels were a positive indicator for increased deposition of AGEs within the arteries, thus, indicating that fetuin-A may indirectly influence the AGE/RAGE pathway especially in the presence of inflammatory molecules.

Fetuin-A (Ahsg) has a high affinity for hydroxyapatite crystals, which are located in sites of vascular calcification, such as bone and teeth [45, 50, 51]. Ketteler et al. utilized patients with CKD on hemodialysis to correlate cardiovascular mortality with decreased fetuin-A levels and increased vascular calcification suggesting that fetuin-A acts as an inhibitor of calcification [6, 52, 53]. Studies using a fetuin-A deficient mice model that were calcification sensitive (DBA/2-Ahsg^−/−^) determined that the glycoprotein is an inhibitor of calcification [54]. X-ray images of the bone and von Kossa staining of the lung, heart, kidney, and skin revealed a visual increase in the deposition of phosphorus and calcium in each tissue type. Blood serum was extracted from DBA/2-Ahsg^−/−^ animals to perform an* in vitro* basic calcium phosphate (BCP) precipitation assay. Fetuin-A decreased the amount of BCP precipitate within the serum, indicating that fetuin-A can inhibit the formation of BCP deposition [54]. Within the same research group, Heiss et al. utilized electron microscopy and dynamic light scattering to determine the structural characteristics of fetuin-A complexing with BCP to form calciprotein particles. Additional studies using purified fetuin-A incubated with BCP* in vitro* resulted in BCP structure changing from a rigid to a fragile appearance [3, 55]. This observed structural change was also observed in other calcium based materials such as CaCO_3_ nanoparticles [56].

The relationship between fetuin-A, BCP, and calcified VSMCs was determined using* in vitro *and* in vivo *HVSMCs model system. Reynolds et al. demonstrated that fetuin-A was localized in the matrix vesicles of calcified HVSMCs in the medial layer of the artery [57]. These calcified HVSMCs were treated with fetuin-A, which inhibited calcium deposition and calcium incorporation in a dose-dependent and cell-mediated manner. VSMCs have been shown to undergo vesicle- and apoptotic body-mediated vascular calcification [58, 59]. Microscopy and western blotting revealed that HVSMC apoptosis was inhibited by fetuin-A. The calcification of released matrix vesicles and apoptotic bodies was quantified by energy dispersive X-ray analysis and showed that fetuin-A also inhibits calcification of these released cell particles. In this same study, it was demonstrated that fetuin-A is an inhibitor of HVSMC calcification mediated by matrix vesicles and apoptotic bodies [57]. In similar studies by Moe et al., fetuin-A was shown to be an inhibitor of calcification in BVSMCs [60, 61]. Taken together, these data demonstrate that fetuin-A is an inhibitor of calcification.

## 5. AGE-RAGE Signaling and Oxidative Stress in Vascular Calcification

The AGE/RAGE signaling cascade has been demonstrated to be akin to a feed-forward loop whereby outcomes such as increased fibrosis, increased RAGE expression, and increase oxidative stressors are produced [62, 63]. Oxidative stress produced by elevated reactive oxygen species (ROS) can disrupt numerous intracellular structures, such as cellular membranes, proteins, lipids, and DNA. ROS products, like hydrogen peroxide, superoxide anions, hydroxyl radicals, and nitric oxide, are generated by mitochondrial oxidases, NADPH oxidases (Nox), and nitric oxide synthases [64]. RAGE activation results in the increased production of ROS by stimulating specific signaling cascades such as TGF-*β*, NF-*κ*B, and Nox-1 [62]. In a study performed by Wei et al., malondialdehyde (MDA) concentration and Cu/Zn superoxide dismutase (SOD-1) activity were used to assess oxidative stress and the ability to initiate a compensatory oxidative stress mechanism in diabetes-mediated vascular calcification animal models. Diabetic animals with VDN-induced vascular calcification had a significant increase in MDA content and significant decrease in SOD activity levels compared to the diabetic group. When isolated VSMCs were treated with increasing levels of AGE, there were elevated ALP activity levels, Nox-1 mediated ROS production, and RAGE expression. Inhibiting RAGE expression consequently decreased ALP activity, calcium content, and Nox-1 protein production while simultaneously increasing SOD-1 levels. Overall, these studies demonstrated that cell isolates from a model diabetes with VDN mediated vascular calcification model were respondent to AGE treatments as evidenced by significantly increased levels of ALP, ROS, Nox-1, and RAGE protein when compared to only diabetic animals [41]. Brodeur et al. utilized a similar animal model to determine if AGEs within an* in vivo* system can be reduced after diabetes-mediated vascular calcification has occurred [65]. Pyridoxamine (PYR), an AGE inhibitor, was administered as a preventive precalcification treatment whereas alagebrium (ALA), an AGE breaker, was given as a therapeutic postcalcification treatment. For these studies, only ALA allowed for a significant reduction in the number of AGEs and calcium content as measured in muscular arteries, such as the femoral artery, but not in larger conducting arteries like the aorta. PYR decreased the overall AGE and calcium levels, but it was not significant in the studied tissues. The difference in effectiveness of both treatments could be due to the mechanisms of action; PYR acts as an AGE preventative whereas ALA acts as an AGE crosslink breaker. The efficacy of several antioxidants therapies, such as alpha-lipioc acid, 4-hydroxy tempol, and apocynin, was also tested. Apocynin treatment resulted in a significant reduction in calcium deposition in the diabetes-mediated vascular calcification animal model. Brodeur et al. demonstrated that a reduction in calcium through targeted ROS antioxidant therapy is a more feasible treatment in an* in vivo *model of vascular calcification [65]. Collectively, these studies demonstrate that the AGE/RAGE cascade is capable of mediating vascular calcification through oxidative stress mechanisms, and therapeutic treatments to limit ROS production might provide a more feasible alternative to minimize vascular calcification.

Another ROS signaling cascade activated by AGEs is transforming growth factor- (TGF-) *β*. In a study by Li et al. when VSMCs were treated with AGEs, members of the AGE/RAGE signaling cascade (i.e., p38 MAPK and ERK1/2) were found to be phosphorylated upon RAGE activation [66]. In addition, TGF-*β* signaling resulted in the phosphorylation of its family of mediators, Smads, which serve as transcriptional modulators [67]. These changes were found to be TGF-*β* dependent. Western blot analysis revealed that when RAGE expression was downregulated, Smad 2 phosphorylation was also inhibited indicating the AGE/RAGE cascade in Smad activation and TGF-*β* signaling. Since the accumulation of AGEs is within the extracellular matrix (ECM), it is important to note that an increase in TGF-*β* has been implicated in fibrosis within disease [68]. Fibrosis is typically associated with an increase in type I collagen and Li et al. utilized western blot analysis to demonstrate that AGEs induce an increased production of type I collagen, which was inhibited by blockade of p38 MAPK and ERK1/2 signaling. These data allow for the conclusion that AGE/RAGE signaling plays a role in the maintenance and regulation of the ECM in diabetes and that AGEs induce TGF-*β* through mediation by RAGE [63, 66, 68].

AGEs have also been shown to increase the activity of NF*κ*B through RAGE signaling in VSMCs. Studies have demonstrated that VSMCs will maintain a compliant, contractile phenotype within the artery; however, increases in NF*κ*B signaling will interfere with this phenotype resulting in increased rigidity and stiffness commonly associated with cardiovascular diabetic complications [69]. Simard et al. treated rat aortic VSMCs (A7r5 cells) with glycated human serum albumin (AGE-HSA) and using GFP expression observed significantly increased NF*κ*B activity. Western blot analysis revealed that ERK1/2 activation was significantly increased with AGE-HSA treatment, and AKT activation was slightly increased. Both of these pathways activate NF*κ*B, which would allow for the conclusion that RAGE signaling increases NF*κ*B activity [69]. An increase in NF*κ*B transcription activity can lead to an increase in mRNA expression of type I collagen a1 and a2 in murine VSMCs treated with AGEs as shown in Peng et al. [70]. Collectively, AGE-induced RAGE signaling affects the activity of NF*κ*B in VSMCs, which can lead to remodeling of the type I collage in the ECM or to a change in cell morphology. Also, when they are treated with AGE-HSA, the mRNA levels of smooth muscle-myosin heavy chair (SM-MHC) and SM-22*α* were decreased, and additionally protein expression of SM-*α*-actin, SM-22*α*, and myocardin (MyoC) was also decreased. Overall, the researchers demonstrated that RAGE signaling interferes with the expression of smooth muscle phenotype markers in A7r5 cells. The loss of smooth muscle phenotype markers offered an explanation for the changes in smooth muscle mechanical cell properties as AGE/RAGE signaling increased. There was also an increased granularity within the A7r5 cells demonstrating a visual change in cell morphology due to increased RAGE signaling. While the overall actin density was unchanged with AGE-HAS treated cells, Young's modulus, a measure of elasticity, revealed that basal cell rigidity was significantly increased indicating a stiffer, less elastic cell type. Protein expression levels of phosphorylated myosin light chain (MLC) were also measured to determine changes in contractile function and actin-myosin-mediated motor activity. These results revealed that no changes in contractile function occurred when A7r5 cells were treated with AGE-HSA. Taken together, increased AGE/RAGE signaling alters the mechanical properties of VSMCs resulting in a stiffer, less compliant cell type.

## 6. Conclusion

**Figure 1:**
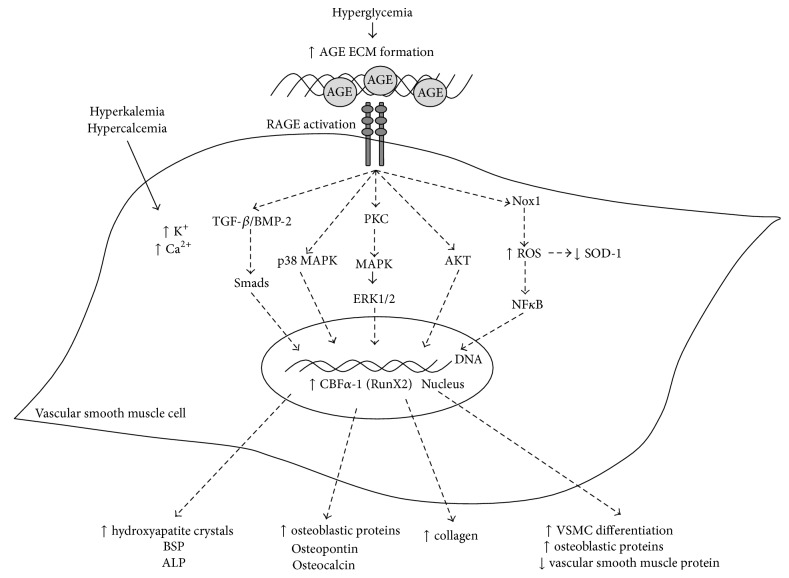
Schematic of AGE/RAGE signaling in diabetes-mediated vascular calcification.

AGE/RAGE signaling is a complex and intricate cascade and has been studied in many different disease states. Particularly, diabetes-mediated vascular calcification exhibits several factors that allows for AGE/RAGE signaling to heavily influence both cellular and systemic responses. Vascular calcification has been demonstrated to increase bone matrix proteins through PKC signaling in hyperglycemic and calcification conditions. AGEs-induced vascular calcification caused downregulation of VSMCs markers and an upregulation of bone matrix proteins, thus, suggesting that the VSMCs undergo a phenotypic switch to an osteoblast-like cell. RAGE signaling can also mediate VSMC calcification through a number of mitogenic pathways. Of those, the p38 MAPK pathway was demonstrated to be an essential component for AGE/RAGE mediated VSMC differentiation. Fetuin A was also shown to play a more controversial role in vascular calcification. Fetuin A acts as a mediator for both procalcification by artificially selecting for AGEs as a RAGE ligand as well as anticalcification in certain models of CDK. Fetuin-A represents an exciting area for more work to be done to understand its role in vascular calcification as a diabetic complication. AGE/RAGE signaling has been implicated in oxidative stress associated with diabetes-mediated vascular calcification through activation of Nox-1, TGF-*β* mediated fibrosis, NF*κ*B, and ERK1/2 pathways and decreased expression of SOD-1. Researchers found that pharmacological agents and certain antioxidants decreased the level of calcium deposition in AGEs-induced diabetes-mediated vascular calcification. Overall, the role of AGE/RAGE signaling in diabetes-mediated vascular calcification was attributed to oxidative stress and the phenotypic switch of VSMCs in AGEs-induced calcification conditions as shown in Figure 1. Future direction in understanding vascular calcification as a diabetic complication could include utilizing RAGE knockout mice to examine the effects of systemic inhibition of RAGE on diabetes-mediated vascular calcification. Also, the role of fetuin-A could be better examined to understand the interplay of this biomarker and AGE/RAGE signaling in type II diabetes.
